# Identification of a Cluster of HIV-1 Controllers Infected with Low Replicating Viruses

**DOI:** 10.1371/journal.pone.0077663

**Published:** 2013-10-30

**Authors:** Concepción Casado, Maria Pernas, Virginia Sandonis, Tamara Alvaro-Cifuentes, Isabel Olivares, Rosa Fuentes, Lorena Martínez-Prats, Eulalia Grau, Lidia Ruiz, Rafael Delgado, Carmen Rodríguez, Jorge del Romero, Cecilio López-Galíndez

**Affiliations:** 1 Virologia Molecular, Centro Nacional de Microbiología (CNM), Instituto de Salud Carlos III, Majadahonda, Madrid, Spain; 2 Laboratorio de Microbiología Molecular, Instituto de Investigación Hospital 12 de Octubre (i+12), Madrid, Spain; 3 IrsiCaixa Foundation, Hospital Universitari Germans Trias i Pujol, Universitat Autònoma de Barcelona, Badalona, Barcelona, Spain; 4 Centro Sanitario Sandoval (CSS), Instituto de Investigación Sanitaria del Hospital Clínico San Carlos (IdISSC), Madrid, Spain; University of Pittsburgh Center for Vaccine Research, United States of America

## Abstract

Long term non-progressor patients (LTNPs) are characterized by the natural control of HIV-1 infection. This control is related to host genetic, immunological and virological factors. In this work, phylogenetic analysis of the proviral nucleotide sequences in *env* gene from a Spanish HIV-1 LTNPs cohort identified a cluster of 6 HIV-1 controllers infected with closely-related viruses. The patients of the cluster showed common clinical and epidemiological features: drug user practices, infection in the same city (Madrid, Spain) and at the same time (late 70’s-early 80’s). All cluster patients displayed distinct host alleles associated with HIV control. Analysis of the virus envelope nucleotide sequences showed ancestral characteristic, lack of evolution and presence of rare amino-acids. Biological characterization of recombinant viruses with the envelope proteins from the cluster viruses showed very low replicative capacity in TZMbl and U87-CD4/CCR5 cells. The lack of clinical progression in the viral cluster patients with distinct combinations of protective host genotypes, but infected by low replicating viruses, indicate the important role of the virus in the non-progressor phenotype in these patients.

## Introduction

Long term non-progressor patients (LTNPs) are individuals infected with HIV-1 for more than 10 years, maintaining high CD4+ lymphocytes numbers without clinical symptoms in absence of therapy [1]. Within the LTNPs group and according to HIV-1 plasma viral load, we can distinguish LTNP non controllers (LTNP-NC) with viral loads above 2000 copies/ml, LTNP viremic controllers (LTNP-VC) with viral load between 50–2000 copies/ml and LTNP elite controllers (LTNP-EC) with undetectable viral loads (<50 copies/ml) [2].

Host genetic, immunological and virologic factors have been investigated in relation to the natural control in HIV-1 LTNPs. Control of viral replication was associated with the presence of certain human leukocyte antigen (HLA) class I alleles, particularly in the HLA-B locus like the HLA-B57/B27 haplotypes with a significantly higher frequency in HIV-1 controller cohorts [3], [4]. Results of genome-wide association studies (GWAS) confirmed only two groups of genetic polymorphisms in the HLA-B and C locus involved in viral replication control [5]–[8]. Qualitative attributes of innate and adaptive immune response was associated with viral control of HIV-1 infection [9], [10]. In this control, the importance of antigen sensitivity and T-cell receptor avidity were reported [11].

Different studies described the correlation between *in vitro* HIV replication and the level of plasma virus load and disease progression in chronic progressors [12] but also in non-progressors patients [13]–[15]. Several studies reported defective virus in LTNP patients [16]–[18] but other works defined the presence of replication competent viruses in HIV-1 LTNP controller patients [19], [20]. Rigorous fitness studies on isolated HIV-1 gene products from HIV-1 controllers indicated that *gag*, *po*l and *env* genes contribute to a reduced replicative fitness [21]–[23]. Except from the Sydney Blood Bank Cohort [24] which included LTNPs patients infected with an attenuated nef/LTR HIV-1 strain, no evidence of a cluster of viruses among HIV-1 LTNP controllers has been described [19], [20].

In this study, we identified by phylogenetic methods, a viral cluster in the sequences from a cohort of Spanish LTNPs. The patients of the cluster shared epidemiological and clinical data supporting the phylogenetic clustering, and they showed host HLA II alleles associated with viral control. In addition, as a first characterization, we analyzed the replicative capacity of recombinant viruses with *env* gene form the cluster patient’s viruses, and identified, in comparison with subtype B sequences, rare amino acids specific of the cluster viruses.

## Materials and Methods

### Ethic Statement

**Figure 1:**
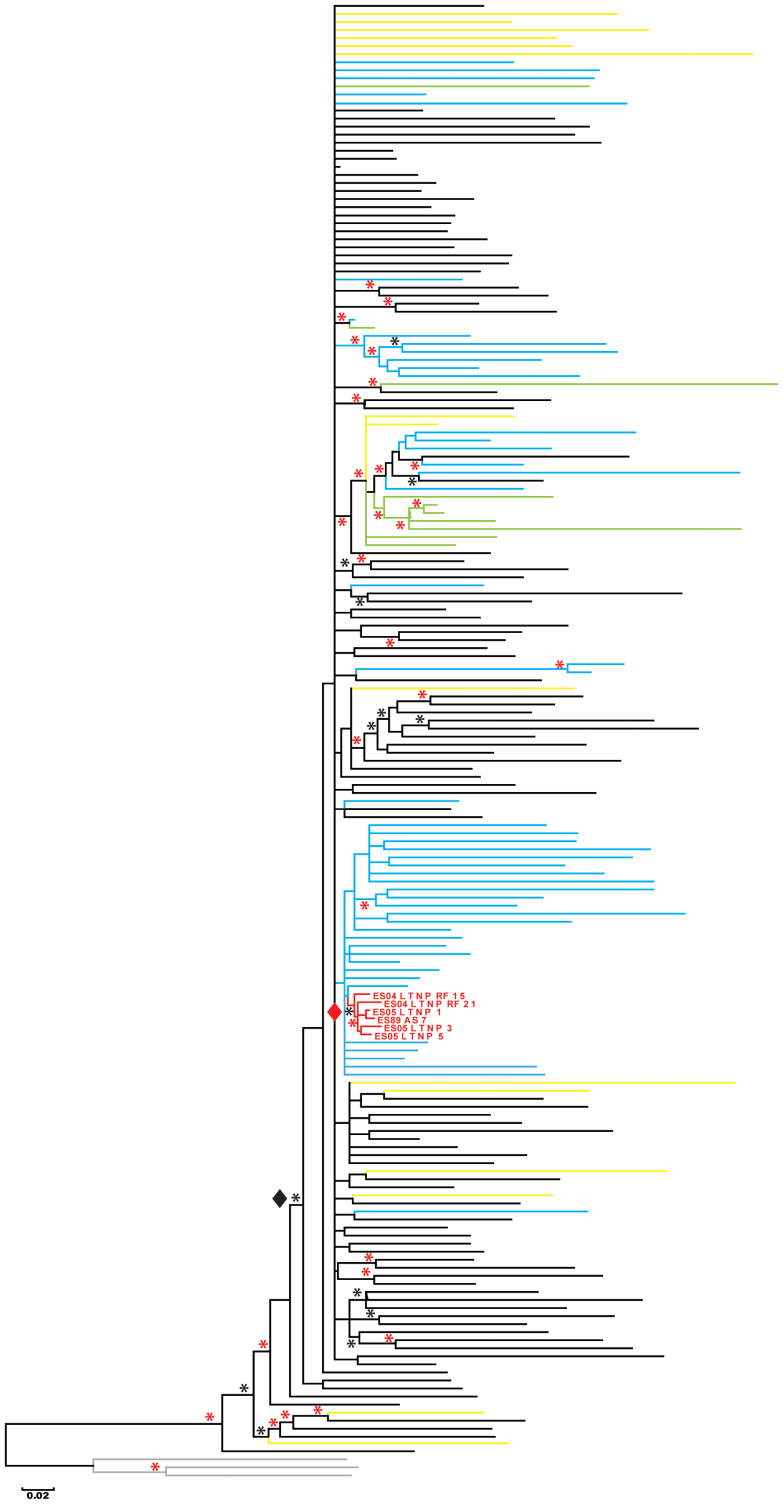
Phylogenetic tree from the Bayesian MCMC (MrBayes) analysis. The 50% majority rule consensus was constructed and posterior probabilities are indicated by asterisks in nodes (black * upper 0.85 and red * upper 0.95). MRCA including the vast of majority sequences analyzed (black ♦) and MRCA from the cluster viruses (red ♦) are marked. Branch lengths represent the mean value observed for that branch among the post-burning sampled trees. The branch colors identified nucleotide sequences origin: black correspond to ancient sequences from North-America (before 1991) and from Europe (before 1995), blue are Spanish sequences (from 1989 and 2005) and red Spanish cluster sequences (from 2004–2005, except As7 which was from 1989). Green sequences are from elite suppressors [54] and yellow sequences from elite controller patients [54]. Gray lines are D subtype sequences used as outgroup in the bottom of the tree.

Participants gave informed consent for genetic analysis studies, which was oral and general for different type of studies for participants with a long term follow-up. Written consent was not obtained from a collection of samples drawn in 1989 from patients without clinical follow-up (included as control Spanish samples in Figure 1 and Fig. S1) because this requirement was not necessary at this time. The remaining samples correspond to patients in follow-up, in which first samples were with oral consent but the written consent was obtained later during the follow-up. The consents were approved by the Ethical and Investigation Committees of the different centers: Centro Sanitario Sandoval (Madrid), Fundació IrsiCaixa (Badalona), Hospital 12 de Octubre (Madrid) and collaborator centers of the HIV BioBank integrated in the Spanish AIDS Research Network (RIS). All clinical investigations were conducted according to the principles expressed in the Declaration of Helsinki. The investigation was approved by the Comité de Ética de la Investigación y de Bienestar Animal of the Insituto de Salud Carlos III with CEI PI 05_2010-v3 number.

### Study Population

Set 1 was formed by 56 HIV-1 complete *env* nucleotide sequences from 55 Spanish patients. This set included 41 LTNPs patients, mostly infected in the 80’s, kindly provided by the Centro de Salud Sandoval, the Fundació IrsiCaixa, the Hospital 12 de Octubre and the HIV BioBank integrated in the Spanish AIDS Research Network (RIS). In addition, we included 3 LTNPs Spanish patients whose nucleotide sequences were deposited in the Los Alamos National Laboratory HIV Database (LANL database, http://www.hiv.lanl.gov) [25]. The remaining 11 samples were collected in 1989 from HIV-1 patients without clinical follow-up. Table S1 summarizes the available clinical and virological characteristics of these individuals.

### Reference Nucleotide Sequences

Set 2 was formed by a group of 128 complete *env* nucleotide sequences from non-Spanish HIV-1 patients selected in LANL database. It included sequences obtained at the beginning of the subtype B HIV-1 epidemic (68 from North-America prior to 1991 and 32 from Europa prior to 1996), 25 sequences from HIV-1 controllers previously described [19], [20] and three subtype D sequences used as outgroup (sample identification and Gene Bank accession number are found in Table S2).

### *Env* Gene Amplification, Nucleotide Sequencing and Phylogenetic Analysis

Complete *env* nucleotide sequences from 39 patients included in the set 1 were obtained from proviral DNA by single genome amplification (SGA) as described [17]. The remaining 16 nucleotide sequences from set 1 were kindly provided by Dr. Delgado (Hospital 12 de Octubre) or obtained from the LANL database. Nucleotide sequences of the non-Spanish group (set 2) were also included in this phylogenetic analysis.

In total, 184 sequences were aligned with CLUSTAL X 2.0 program [26], and Gblocks program [27] was used to eliminate poorly aligned positions, primarily in the variable regions. Phylogenies were estimated using a classical and a Bayesian approach, both functioning under a maximum likelihood (ML) criterion and without assuming any molecular clock. The classical approach was implemented using the best-fit model of nucleotide substitution (GTR+G+I, jModelTest v.0.1.1) [28] in PhyML 3.0 program [29] in order to calculate the ML tree. Internal branches support was tested with an approximate likelihood-ratio test [30].

The Bayesian approach was implemented by using MrBayes v.3.2 [31] with the GTR+G+I model of nucleotide substitution. Four independent MCMCMC (Metropolis-Coupled Markov Chain Monte Carlo) runs starting from a random tree were calculated, each for 3×10^7^ generations, with a burn in of 6×10^6^ generations from each run. Examination of MCMCMC samples with Tracer 1.3 [32], indicated the adequate mixing of the MCMCMC. Phylogenetic trees were visualized using FigTree v.1.3 (http://tree.bio.ed.ac.uk).

Most recent common ancestor (MRCA)-to-tip distances were extracted from MrBayes phylogenetic tree using TreeStat v.1.2 (http://www.tree.bio.ed.ac.uk/software/treestat). Because no molecular clock was assumed for the analysis, we tested the correlation between genetic distances to the MRCA and sampling time of nucleotide sequences obtained at the beginning of the HIV-1 epidemic (years 1981–1995). This correlation was used to estimate the “viral dating” of the nucleotide sequences from the cluster viruses.

### Complete *env* Gene Sequences Analysis

The amino acid sequences of HIV-1 cluster were compared to consensus B sequence obtained from LANL database. Each amino acid position modified in at least 5 sequences from the cluster viruses was compared to the ancient (110 nucleotide sequences prior to 1996 from set 1 and 2) and Spanish (50 sequences from set 1) groups in our alignment and with the complete *env* subtype B amino acid sequences included in the 2006 web alignment of the LANL database (452 sequences). For the comparison of the cluster sequences with each set of sequences, a statistical analysis was performed with the non-parametric Mann-Whitney U test with a restrictive significance at the 99.9%.

We also compared the gp160 total length and the length variation in the gp120 loops (number of amino acid between cysteine residues), V1/V2 (amino acid 130 to 190 in subtype B consensus), V3 (amino acid 293 to 327), V4 (amino acid 380 to 405), V5 (amino acid 447 to 456) and in the gp120 signal peptide (SP) (amino acid 1 to 34). Potential N-linked glycosylation sites (PNLGS) were also predicted using the N-GLYCOSITE web tool (http://www.hiv.lanl.gov content/sequence/GLYCOSITE/glycosite.html).

### Virus Isolation from Patient’s Peripheral Blood Mononuclear Cells (PBMC)

PBMC and plasma samples were obtained from the patients as described [33]. Virus isolation from purified CD4^+^ T cells was attempted by co-culture as previously described [17]. Co-cultures were maintained for one month and tested for p24 production by the electrochemiluminescence Immunoassay ECLIA using Elecsys 2010 immunoassay analyzers (Roche Diagnostic).

### Generation of Recombinant Viruses

Full-length *env* genes from three patients of the cluster (LTNP_1, LTNP_3 and LTNP_RF_21), from three HIV-1 infected chronic progressors (I10 and IV10 obtained in 1993 from IDU patients and RIS06) and one laboratory adapted virus (SF-162) were cloned into 89ES061 molecular clone, derived from a Spanish field isolate [34]. Molecular clone 89ES061 was *SapI* digested, gel extracted using PureLink Quick Gel Extraction Kit (following the manufacturer instructions, Invitrogen) and ligated with the gp160 amplicons using T4 DNA ligase (New England Biolabs). Recombinants plasmids were transformed in DH5α competent cells and clones sequenced to check the correct insert orientation.

In order to generate the recombinant viruses, 20 µg of the recombinant plasmids were transfected into 3×10^6^ 293T cells using a calcium chloride protocol [35]. 293T cells were maintained in Dulbecco’s modified Eagle medium (DMEM) supplemented with 10% fetal bovine serum, 2 mM L-glutamine, 100 U/ml penicillin and 100 µg de streptomycin/ml (DMEMc). 72 h post-transfection, supernatants were harvested and filtered through 0.45 µm to remove cellular debris. Virus production was quantified measuring in the supernatants HIV-1 p24 antigen by electrochemiluminescence Immunoassay (Roche Diagnostic) and reverse transcriptase (RT) activity using a Syber green I-based real-time PCR enhanced RT assay (SGPERT) [36].

### Viral Titer Determination

Recombinant virus titration was performed in 10^4^ TZM-bl cells with 100 µL of serial 10-fold dilutions of viral stocks; each dilution was assayed six times. After 48 h, cells were stained for β-galactosidase activity as described [37]. Titers represented the mean and standard error of three independent assays and they were expressed in tissue culture infective dose per ml (TCID/ml), calculated by the Spearman–Karber formula [38].

### Replicative Capacity of Cluster Viruses

Viral infectivity of recombinant viruses was assayed in duplicate by infection of 10^5^ TZM-bl cells with fifteen p24 antigen units of recombinant viral stocks, corresponding to 75 pg, in the presence of 40 µg/mL of DEAE-dextran. After 48 hours of incubation, infection levels were determined by luciferase activity of cell lysates using the luciferase assay system (Promega). As negative control luciferase activity from a transfection supernatant without plasmid was used. Luciferase activity values were normalized to the luciferase activity of wild-type (WT) virus (89ES061 clone).

Replicative capacity of recombinant viruses was also assayed by infection of U87-CD4/CCR5 cells. U87-CD4/CCR5 cells were cultured in DMEM supplemented media with 15% fetal bovine serum plus 300 µg/ml G418 (Sigma-Aldrich) and 1 µg/ml puromycin (Sigma-Aldrich). 5×10^4^ U87-CD4/CCR5 cells per well were seeded in a 24 well plate and infected with 100 units (corresponding approximately to 500 pg) of p24 antigen of the different viruses. Virus production was quantified by measuring the RT-activity in the supernatants. Cultures were sub-cultured during 14 days.

### Characterization of Genetic Markers of the Patients

The host genetic polymorphisms studied were chosen on the basis of GWA studies or selected from the literature [2]. These included the *HCP5* rs2395029 allele in linkage disequilibrium with HLA-B*5701, the *HLA-C-35* (rs9264942) variant, *CCR5* Δ*32* (rs333), *CCR2 V64I* (rs1799864), *ZNRD1* (rs3869068) and *HLA-class I (A, B, C)*.

### Nucleotide Sequence Accession Numbers

The new sequences reported in this paper have been deposited in the GeneBank database (accession numbers: KC595149-KC595178, KC595182-KC595203 and KC595221).

## Results

### Identification of a Viral Cluster in a Spanish HIV-1 LTNPs Cohort

The global and quasispecies nucleotide sequences from a cohort of Spanish LTNPs and chronic patients, obtained between 1989 and 2005, were analyzed by Maximum Likelihood (ML) in the gp120 C2-V5 region in *env* gene (Figure S1). This analysis showed that all nucleotide sequences obtained in different time points from each patient formed monophyletic clades, except sequences from 6 individuals. These sequences could not be segregated per patient, and they formed a phylogenetic cluster with short branch lengths and very low mean genetic distances among sequences (1.1%) although samples were recovered during 16 years (1989–2005). Viral dating of the nucleotide sequences from the cluster viruses, according to the methodology described for Spanish viruses [39] indicated the ancestral characteristics of the virus (Table 1), i.e. viral dating close to the time of primoinfection [39]. All patients included in the cluster had intravenous drug practices (IDU) in the late 70’s and early 80’s; they lived in Madrid and had a first HIV-1 positive serology between1985 and 1990 (Table 1). Clinical and virological follow-up, for over 15 years, was only possible in five of the six patients. Four of them were LTNP-EC (LTNP_3, LTNP_5, LTNP_RF_21 and LTNP_RF_15) and one was a LTNP-VC (LTNP_1) (Table 1 and Figure S2) [2].

**Table 1:** Epidemiological, clinical and virological characteristics of the cluster patients.

Patient	BirthDate	Sex	Origin	Hospital	Riskgroup	IDUyears	FirstHIV-1+	Viraldating^a^	Mean	Mean CD4^+^	Mean CD8^+^	HVCGenotype
									V.LoadCopies/ml	Cells/µl	Cells/µl	
LTNP 1	1954	F	Madrid	C.S.Sandoval	IDU	1978–80	1990	Ancestral	285	745	1333	1a/1b
LTNP 3	1956	M	Madrid	C.S.Sandoval	IDU	1976–82	1988	Ancestral	<50	775	570	1b
LTNP 5	1962	M	Madrid	C.S.Sandoval	IDU	1980–88	1986	Ancestral	210	876	1302	1a
89 AS7	1965	F	NK	El Patriarca	IDU	NK	1989	Ancestral	NK	NK	NK	NK
LTNP RF 15	1964	M	Madrid	12 de Octubre	IDU	1985–94	1989	Ancestral	<50	478	810	3
LTNP RF 21	1961	M	Madrid	12 de octubre	IDU	1977–99	1985	Ancestral	<50	797	908	1

a according to [21].

NK: not known.

Except for LTNP_RF_21 (Table 2), all patients showed an HLA-B haplotype associated with viral control (B27**, B57**/B58** and B51**) and all individuals presented an accumulation of genetic polymorphisms *HLA-C-35* (rs9264942), *CCR5* Δ*32* (rs333) and *CCR2 V64I* (rs1799864) associated with lack of clinical progression (Table 2). In summary, we detected in a cohort of Spanish HIV-1 LTNP patients, a cluster of epidemiologically linked individuals, with genetic factors associated with the control of HIV-1 replication, and infected with closely-related ancestral viruses.

**Table 2:** Host genetic factors of the cluster patients.

Paciente	CCR2 V64Irs1799864	CCR5 del32rs333	HCP5rs2395029	HLA-Crs9264942	ZNRD1rs3869068	HLA-A		HLA-B		Score^a^
LTNP_1	11	11	11	**12**	11	0201	2401	1501	**2705**	2
LTNP_3	11	11	11	***22***	11	0201	0205	**2705**	**5801**	4
LTNP_5	**12**	**12**	11	***22***	11	0201	6801	**2705**	*3503*	4
AS7	**12**	11	11	**12**	11	0301	6901	3501	**5101**	3
LTNP RF 15	11	11	ND	11	ND	02	24	44	57	1
LTNP RF 21	**12**	11	ND	***22***	ND	ND	ND	47	14	3

For SNPs, 1 indicates the most common and 2 the variant allele. Protective HLA alleles included B*2705, B*5701, B*5101, B*1302, A10 serogroup (A*2501, A*2601) and A*3201. Risk HLA alleles included B*35Px (B*3503), B22 serogroup (B*55, B*56), B*1801, A*2402 and A*2301.

a Additive unweighted genetic scores were used to compile genetic information: *CCR2 V64I* [0,1,2], *CCR5 Δ32* [score 0,1], *HLA-C–35* [0,1,2], and protective *HLA-B+* [0,1,2] and detrimental *HLA-B-* alleles [0,−1,−2]) according to [1]: 0: regular font, +1: bold, +2: bold and italic and −1 italic.

### Phylogenetic Confirmation of the Viral Cluster

For the clarification of the phylogenetic history of the cluster viruses and the exclusion that clustering was due to the ancestral characteristic of the viral nucleotide sequences, we extended the phylogenetic study to the complete *env* gene and to nucleotide sequences from the beginning of the HIV-1 epidemic. For this analysis, we used only one sequence from set 1 and set 2 of nucleotide sequences (Materials and Methods and Table S1 and S2). As only 11 Spanish nucleotide sequences prior to 1996 could be obtained, we analysed nucleotide sequences from Spanish LTNPs, obtained between 1998 and 2005, but infected in the 80 s and early 90 s (see Table 1). The phylogenetic trees obtained by ML (data not shown) and Bayesian approach (Figure 1) identified the same viral cluster than in the C2-V5 fragment (Figure S1). The posterior probability for the viral cluster was 0.87 and it increased to 0.98 when patient LTNP_RF_15 was not considered.

Another striking characteristic of the viral cluster was the very low genetic distance to the most recent common ancestor (MRCA), marked in Figure 1, which includes the majority of the samples analyzed. Except for three samples, the mean genetic distance of the cluster to the MRCA was among the shortest in the tree with a value of 4.85%. This value demonstrated the proximity of the viral cluster to the origin of the subtype B epidemic in the developed countries.

**Figure 2:**
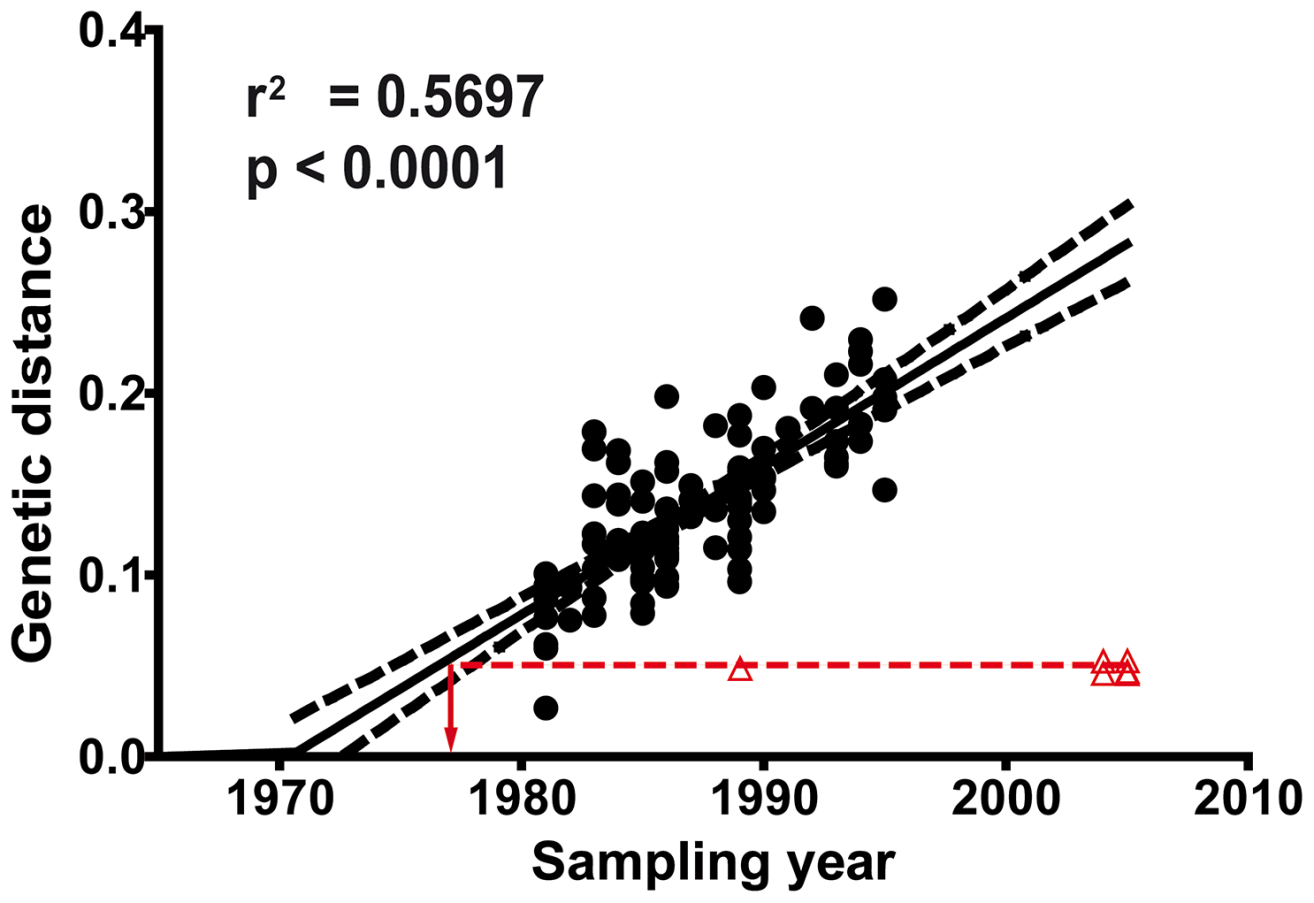
Correlation between sampling time and the genetic distance with reference and viral cluster strains. MRCA-to-tip distances were extracted from MrBayes phylogenetic tree using TreeStat v.1.2. •, values obtained for the nucleotide sequences collected at the beginning of HIV-1 epidemic (years 1981–1995) were plotted against sampling time and a linear regression analysis was performed. Red Δ, values obtained from the cluster nucleotide sequences. The red dashed line and the arrow permit the extrapolation of the year from cluster nucleotide sequences.

The Bayesian inference was done with no molecular clock assumption and then it was possible to test the correlation between the genetic distance to the MRCA and the sampling year for the HIV-1 sequences collected at the beginning of subtype B epidemic in developed countries (years 1981–1995) (Figure 2). In spite of scattered data, a good correlation was obtained between genetic distance and sampling time (r^2^ = 0.57, p-value <0.0001) and with a divergence rate of 0.81%/year. Although samples from the cluster patients were collected between 2004 and 2005, the extrapolation of the genetic distance to the MRCA allowed the viral dating of the cluster samples at around 1977–1978, thus confirming the ancestral characteristics. The low genetic distance to the MRCA and the ancestral viral dating, implying 27–28 years of infection, confirmed the lack of viral evolution in these patients.

### *Env* Gene Length and Potential N-linked Glycosylation Sites in the Viruses of the Cluster

**Figure 3:**
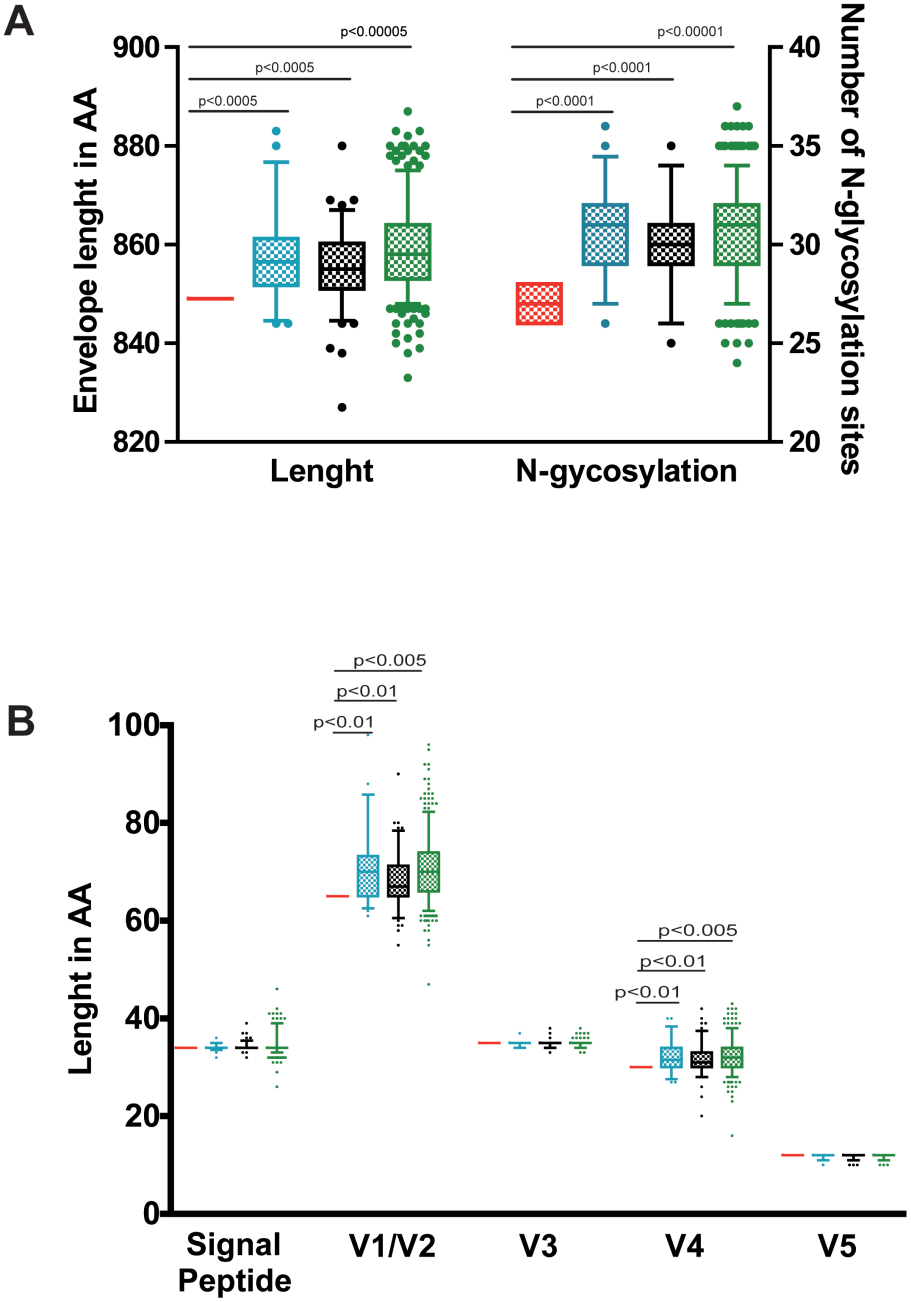
Envelope analysis of the cluster viruses. A) Envelope length in amino acids and N-linked glycosylation sites (NxT/S). B) V1 to V5 loop and signal peptide (SP) lengths in the amino acid sequence sets analysed. Red cluster nucleotide sequences, blue Spanish nucleotide sequences, black ancient nucleotide sequences and green subtype B nucleotide sequences. *P* values for comparison between 2 groups shown with horizontal black bars were calculated using a 2-tailed Mann-Whitney test.

The total *env* gene length, the V1 to V5 loop and signal peptide (SP) lengths and the number of potential N-linked glycosylation sites (PNLGS) in the cluster sequences were compared to three sequence groups: the 2006 reference alignment from the LANL database (454 subtype B sequences), the 110 ancient sequences group (obtained prior to 1996 from set 1 and set 2) and the 50 Spanish sequences group (set 1) from our alignment. The gp160 protein total length (849 amino acids) and the number of PNLGS (26–28 sites) from the cluster nucleotide sequences were very similar in all patients confirming infection with closely-related viruses. These values were significantly lower (p<0.0005) than those in the three control sets (Figure 3A). Analysis of the gp120 loops and the SP lengths revealed that the differences in total length were attributed to significant differences in the V1–V2 and V4 regions (Figure 3B). The identical positions of the PNLGS support the relatedness of the viruses, and the short length of the variable loops the ancestral dating of the cluster viruses.

### Biological Characterization of Viruses from the Cluster

For the identification of viral factors potentially implicated in the lack of evolution detected in these patients, virus isolation was attempted from purified CD4^+^ T cells at least twice in three cluster patients (LTNP_1, LTNP_3 and LTNP_5). All co-cultures were negative, except for one LTNP_1 sample. In this case, although co-culture supernatant was antigen p24 positive, we were unable to propagate the virus in donor peripheral blood mononuclear cells (PBMCs).

Since we could not isolate the viruses, as a first characterization of the biology of the viruses, we analyzed recombinant viruses with the *env* gene from proviral DNA of patients LTNP_1, LTNP_3 and LTNP_RF_21. This cloning was carried out into the laboratory infectious clone 89ES061 from a natural Spanish isolate (see Materials and Methods). We also obtained recombinant viruses from three control HIV-1 infected chronic progressor patients (I10, IV10 and RIS06) and one laboratory adapted CCR5-virus (SF-162) and they were assayed in different cell lines.

To perform the biological characterization of the recombinant viruses obtained by transfection in 293T cells, p24 antigen, RT- activity and TCID_50_ were determined in the transfection supernatant and the results are shown in Table 3. We found a direct correlation between the p24 level and the RT-activity (R^2^ = 0.96, p<0.0001 Pearson correlation) but not with the infectious titer. Although lower values were observed in p24 antigen levels and RT- activity, no large differences between the cluster viruses and the other viruses, except from virus from patient LTNP_RF_21, were detected. All recombinant viruses from the cluster showed low infectivity titers, with a maximum of 6.2×10^2^ TCID_50_, which are 10 to 100 times lower than control viruses. The titer/p24 antigen ratio from cluster recombinant viruses was between 0.22 and 2.73; ratio from chronic viruses ranged between 10.89 and 21.29, while the ratio of the WT and the SF-162 reference viruses was 17.13 and 94.16 respectively. Then, the cluster viruses showed titer/p24 antigen ratios that were from 6–78 folds lower than those of WT virus and 34–428 lower than SF-2 virus.

**Table 3:** Biological characterization of recombinant viruses with *env* genes from the cluster patients.

	p24±SE^a^	RT±SE^b^	TITER±SE	TITER/p24
	U/ml	UA/ml	TCID_50_/ml	TCID_50_/U
*LTNP_1_12*	*(3,2±0,48)×10^2^*	*(4,7±0,04)×10^2^*	*(6,2±2,01)×10^2^*	*1.95*
*LTNP_1_10*	*(5,3±0,75)×10^2^*	*(5,8±1,27)×10^2^*	*(0,62±0,10)×10^2^*	*0.12*
*LTNP_3_20*	*(2,5±0,43)×10^2^*	*(4,3±0,22)×10^2^*	*(0,57±0,21)×10^2^*	*0.22*
*LTNP_3_22*	*(1,7±0,32)×10^2^*	*(2,21±0,15)×10^2^*	*(4,7±1,98)×10^2^*	*2.73*
*LTNP_3_3*	*(2,4±0,54)×10^2^*	*(3,13±0,20)×10^2^*	*(2,0±0,58)×10^2^*	*0.85*
*LTNP_RF_21*	*(0,27±0,03)×10^2^*	*(0,36±0,04)×10^2^*	*(0,39±0,16)×10^2^*	*1.46*
I10_28	(1,3±0,15)×10^3^	(2,2±0,03)×10^3^	(1,44±0,35)×10^4^	10.88
IV10_5	(5,1±0,55)×10^2^	(7,6±0,04)×10^2^	(6,79±0,01)×10^3^	13.36
RIS06_2	(1,1±0,13)×10^3^	(1,9±0,01)×10^3^	(1,15±0,15)×10^4^	10.89
SF_162	(3,5±0,34)×10^2^	(5,4±0,02)×10^2^	(3,27±1,47)×10^4^	94.16
WT	(6,1±0,80)×10^2^	(8,9±1,07)×10^2^	(1,04±0,47)×10^4^	17.13

a p24 HIV Ag was determined by the electrochemiluminescence Immunoassay ECLIA using Elecsys 2010 immunoassay analyzers (Roche Diagnostics). Quantitative measure indicated that 10 U/ml corresponded to 50 pg/ml of p24 HIV Ag.

b Reverse transcriptase activity was quantified using in-house Syber green I- based real-time PCR enhanced RT assay (SGPERT). 1 UA/ml corresponds to the RT-activity obtained from a 10^−6^ virus dilution of a viral stock with 10^6^ TCID50/ml.

Italic font: recombinant viruses from the cluster patients.

**Figure 4:**
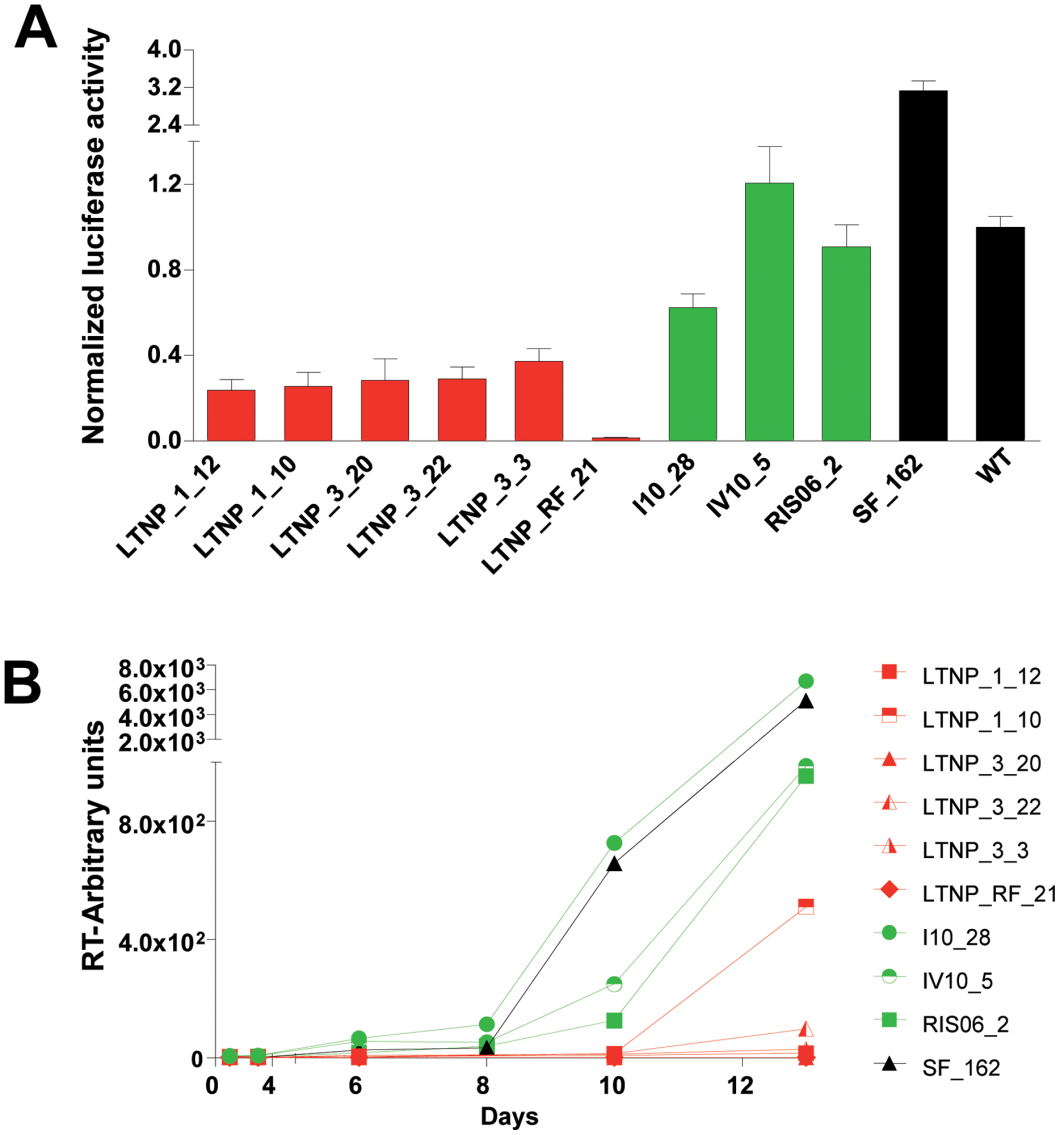
Biological characterization of the recombinant viruses. A) Infectivity of *env* recombinant viruses in TZM-bl cells. Cells were infected with 15 units of HIV-1 p24 antigen (75 pg) of virus-supernatants. Luciferase activity was measured 48 hours post-infection and the results were normalized to the value obtained with the WT virus (89ES061). Results represented the median and SE of two independent assays with thee replicates. B) Replication kinetics of *env* recombinant viruses in U87-CD4/CCR5 cells. Cells were infected with 100 units of HIV- 1 p24 antigen (500 pg) of virus supernatants. Cultures were followed during 14 days, and HIV-1 production was quantified by the RT-activity in the supernatant with in-house Syber green I based real-time PCR enhanced RT assay (SGPERT). Cluster’s recombinant viruses (red) were compared with recombinant viruses from chronic progressor patients (green), with a recombinant virus obtained from laboratory strain SF-162 (black), and with the laboratory infectious clone 89ES061 (black) where the nucleotide sequences were cloned.

Infectivity of the cluster and chronic recombinant *env* clones was also tested by measuring luciferase activity in TZM-bl cells. For these experiments, TZM-bl cells were infected with a fixed amount of antigen p24 from the transfection supernatants (see Materials and Methods). Again, the luciferase activity from all recombinant viruses from the cluster patients was significantly decreased, with values ranging from 0 to 40% those of the WT virus (Figure 4A).

Finally, the recombinant viruses were used for infection of U87-CD4/CCR5 cells. In this experiment, cells were infected with 100 units of p24 antigen (500 pg) of recombinant viruses. Cultures were followed during 14 days, and HIV-1 production quantified measuring RT-activity in the supernatant. In this case, as the WT virus could not be used as control because it is a CXCR4 virus, we employed the laboratory adapted SF-162 virus. The replication curves are shown in Figure 4B. The replication kinetics of the cluster viruses were clearly retarded relative to chronic patient or control viruses. Only two (LTNP_1_10 and LTNP_3_22) of the six clones from the cluster patients reached detectable but low replication levels (with a maximum of 5.0×10^2^ arbitrary units, AU) which are at least 10 times lower than the control values. In addition, two other cluster viruses (LTNP_1_12 and LTNP _3_3) showed extremely low levels (15 and 28 AU respectively) of viral replication only 14 days post-infection. Finally, the other two cluster viruses did not replicate (LTNP_3_20 and LTNP_RF_21). All these results indicated that the cluster viruses were low and slow replicating viruses.

### *Env* Amino-acid Analysis in the Cluster Viruses

**Figure 5:**
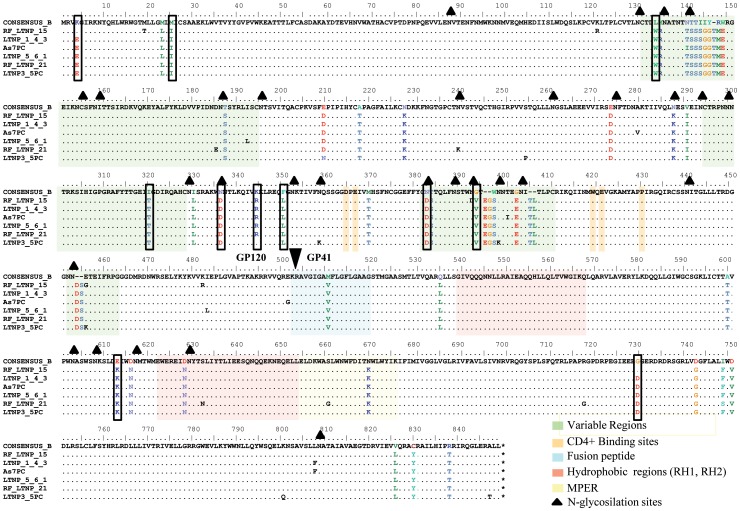
Comparison of the *env* gene amino acid sequences derived from cluster viruses with subtype B consensus sequence. 35 common mutated positions detected in at least 5 of the cluster viruses are shown in color amino acids. Boxes marked the unusual amino acid whose presence in the cluster is statistically significant when compared with the reference amino acid sequence sets used in the study (see Table 3).

Once the existence of the cluster viruses and the low replicative capacity of the recombinant viruses with the envelopes from the patients were confirmed, we investigated the amino acid that could be associated with this phenotype. For this reason, we compared the *env* amino acid sequences of the cluster viruses with the subtype B consensus sequence obtained from the LANL database (Figure 5). Mutations, insertions and/or deletions in the envelope variable regions V1 to V5 were not included in this analysis. We detected the presence of 35 mutated positions common to at least 5 of the 6 viruses. We determined how often these 35 positions were altered in the three sample groups described in *env* length and PNLGS paragraph (2006 LANL database reference alignment, the ancient sequences group and the Spanish sequences group). This analysis permitted the removal of positions in the cluster viruses that could represent ancestral characters or a founder effect in the Spanish HIV-1 epidemic. The presence or absence of every cluster virus mutation into each set of sequences was compared using the non-parametric Mann-Whitney U test. This analysis reduced the number of potential characteristic mutations from 35 mutations to 11, which are highlighted in Table 4. In previous studies, the identification of signature amino acids in viruses from HIV controller patients was associated with loss of replicative capacity and fitness [13], [20], [23], [40].

**Table 4:** Identification of amino acid positions associated with the cluster viruses.

Protein	Codon	N° of Clustersequences withpolymorphism	N° of Spanishsequences withpolymorphism	*P* value	N° of Ancestralsequences withpolymorphism	*P* value	N° of Subtype Bsequences withpolymorphism	*P* value
		(N = 6)	(N = 50)		(N = 110)		(N = 452)	
**GP160**	***K4E***	***5***	***0***	***3,08E-08***	***1***	***2,36E-09***	***1***	***5,50E-13***
	M23L	6	28	0,071	*8*	*1,01E-06*	*32*	*2,17E-07*
	***M25I***	***6***	***4***	***6,47E-06***	***1***	***2,36E-09***	***5***	***3,63E-11***
	***L133W***	***6***	***10***	***2,47E-04***	***2***	***9,44E-09***	***25***	***5,78E-08***
	M134R	6	14	1,19E-03	*27*	*3,73E-04*	*46*	*1,60E-06*
	T186S	6	ND	ND	ND	ND	*60*	*7,13E-06*
	E208D	5	16	0,023	*2*	*7,74E-07*	*10*	*1,05E-07*
	A216T	6	15	1,67E-03	*32*	*9,30E-04*	*92*	*8,27E-05*
	N226K	6	18	4,15E-03	*31*	*7,83E-04*	*83*	*4,56E-05*
	E272D	6	14	1,19E-03	*29*	*5,47E-04*	*62*	*8,59E-06*
	N286K	6	18	4,15E-03	*31*	*7,83E-04*	*95*	*9,95E-05*
	V289I	6	24	0,025	*9*	*1,69E-06*	*38*	*5,54E-07*
	***I319T***	***6***	***3***	***2,59E-06***	***2***	***9,44E-09***	***20***	***1,81E-08***
	I329L	6	18	4,15E-03	50	0,010	164	2,41E-03
	***N335D***	***6***	***7***	***5,29E-05***	***6***	***3,11E-07***	***21***	***2,32E-08***
	***K343R***	***5***	***4***	***1,85E-04***	***5***	***9,07E-06***	***25***	***4,86E-06***
	***F349L***	***6***	***10***	***2,47E-04***	***2***	***9,44E-09***	***8***	***2,36E-10***
	M368T	6	16	2,30E-03	*30*	*6,56E-04*	*73*	*2,018-05*
	***N381D***	***6***	***5***	***1,42E-05***	***11***	***4,17E-06***	***55***	***4,36E-06***
	T382S	6	29	0,073	61	0,038	*138*	*8,75E-04*
	***G392V***	***6***	***5***	***1,42E-05***	***10***	***2,70E-06***	***32***	***2,17E-07***
**GP41**	M7V	6	26	0,032	*27*	*3,73E-04*	*98*	*1,19E-04*
	Q32L	6	31	0,085	55	0,028	151	1,48E-03
	A96T	6	24	0,025	53	0,027	*137*	*8,39E-04*
	***E110K***	***6***	***12***	***5,72E-04***	***31***	***7,83E-04***	***54***	***3,93E-06***
	D113N	6	17	3,11E-03	44	5,36E-03	160	2,08E-03
	D125N	6	17	3,11E-03	35	1,52E-03	*80*	*3,69E-05*
	N166K	6	29	0,073	*24*	*2,00E-04*	*106*	*1,88E-04*
	***G226D***	***5***	***6***	***6,55E-04***	***2***	***7,74E-07***	***3***	***1,99E-09***
	D239G	6	18	4,15E-03	*4*	*7,08E-08*	*13*	*2,13E-09*
	I245F	5	27	0,223	29	8,14E-03	*56*	*1,91E-04*
	D247V	6	30	0,078	52	0,027	213	0,011
	V322L	6	40	0,352	46	6,86E-03	165	2,50E-03
	C326Y	6	18	4,15E-03	34	1,29E-03	***81***	*3,97E-05*
	R334T	6	30	0,078	44	5,36E-03	156	1,80E-03

AA positions characteristics of the viral cluster that show a statistical significance at the 99.9% level within each sequence set are shown in italic.

AA positions characteristic of the viral cluster with a statistical significance at the 99.9% level simultaneously in the three sequence sets are shown in bold and italic.

Nine of these mutations were located in the gp120. Six of them (I319T, N335D, K343R, F349L, N381D and G392V) mapped to the V3-C4 region, two mutations (K4E and M25I) correspond to the SP and one to the V1 region (L133W). The two gp41 mutations were E110K, next to the gp41 immunodominant C-C loop, and G226D in the Kennedy epitope in the cytoplasmic tail. The statistical analysis performed in the viral cluster, identified 11 unusual amino acids that could be related to the lack of viral and clinical evolution in the patients.

### Presence of the Cluster Specific Residues in Functional Viruses

**Figure 6:**
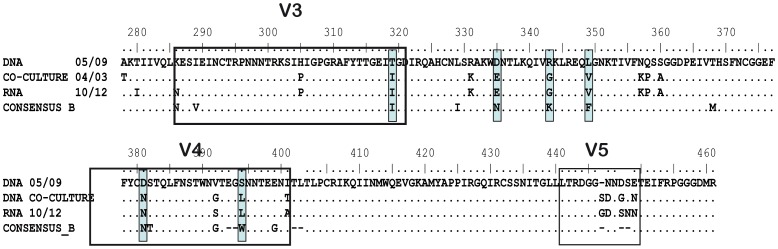
C2-V5 nucleotide sequences from patient LTNP_1. LTNP_1 samples were obtained from PBMC DNA (2005 sample), cell-free supernatant of PBMC co-culture from a 2004 sample and plasma RNA corresponding to a 2010 sample. Shaded boxes indicated amino acid mutations characteristics of the cluster viruses in this C2-V5 region. V3, V4 and V5 regions are indicated by boxes.

The role of the residues, identified by the statistical analysis, in the replicative characteristics of the viruses was confirmed in comparison with functional viruses derived from the cluster patients. To this end, we used viral nucleotide sequences from the viremic patient LTNP 1. LTNP 1 was the only patient in which we obtained a positive co-culture in one of the samples. The virus from the supernatant of this co-culture was partially sequenced in the C2-V5 region in *env* gene. In addition, this patient after 20 years of infection showed an increase in the plasma viral load (Figure S2). A sample from this blip was obtained and the plasma RNA sequenced. These two sequences, which could be considered to be from replicating viruses, are shown in comparison with the sequence from the 2005 proviral sample in Figure 6. These two replicating viruses from LTNP 1 changed the six positions identified by the statistical analysis in this fragment (see Table 4 and Figure 6). In two positions the replicating viruses recovered the amino acid present in subtype B consensus (I319, N381). In the other positions, the changes observed E335, G343, V349 and S392 introduced very unusual amino acids of the subtype B sequences of LANL database (frequency of 0.066, 0.026, 0.004 and 0.083 respectively). In addition to these variations, the replicating viruses also included other changes, giving net electrical charge, volume or structural alterations, like N357K, Q358P and S360A in the C3 region close to the CD4 binding site (364DPE367), which could also affect to the replication capacity of the viruses. In summary, the modification of the six residues in the C2-V4 region, specific for the viral cluster, in the replicating viruses from one of the cluster patients support the deleterious role of these residues in viral replication.

## Discussion

This work provided evidence that within a cohort of HIV-1 Spanish LTNP, a group of LTNP-controllers were infected by closely related ancestral virus. The viral cluster was supported by two independent phylogenetic approaches, ML and Bayesian inference, both assuming no molecular clock. The existence of an HIV-1 cluster in LTNP controller patients has not been previously described in similar cohorts [19], [20], [41]. We showed that the lack of clinical progression of the cluster patients could be related to infection with low replicative viruses.

The objective of the nucleotide analysis was the phylogenetic reconstruction more than the time frame establishment in the phylogeny. Consequently, we did not use any molecular clock approach in the analysis. In both phylogenetic reconstructions, although samples were collected in 2004–2005, the position of the cluster nucleotide sequences in the phylogenetic trees, close to the ancestral nodes of the tree, highlights the ancestral characteristics of the viral cluster. Viral dating of the sequences directly from the phylogenetic reconstruction (Figure 2) showed that the virus of these patients corresponded to the end of 70’s (see Table 1 and Figure 2). Epidemiological data supported a linkage between the cluster patients because of IDU practices in the same city (Madrid, Spain) and in the same period of time. The clustering of the sequences could not be the consequence of the same geographical, temporal origin or risk group, because the other 11 sequences from IDU patients obtained in Madrid in 1989 and included in Figure 1, were not contained within in the cluster (Figure 1 and Figure S1).

Gilbert et al. [42] employing many of the samples included in our set 2 estimated the timing of MRCA of HIV-1 subtype B in the U.S. epidemic to 1969 (1966–1972). Viral dating of the cluster nucleotide sequences, shown in Figure 2, was around 1977–1978 suggesting that its introduction in Spain occurred at the beginning of the HIV-1 epidemic. This assumption is supported by the epidemiological data which reported the first AIDS case in Spain in 1981.

After 27–28 years of infection according to this viral dating and the timing of the cluster samples (2004–2005), a puzzling characteristic of the cluster viruses is the extremely low intra-cluster mean genetic distance (0.8%) (Figure 1). This result establishes a rate of viral evolution of 0.029% nucleotide substitutions by year and reflects the extremely limited viral replication produced in these patients. These minimum distances are even lower than between HIV-1 isolates obtained close to the transmission event in transmission cases [43]–[45]. This lack of viral evolution is also supported by the position of the cluster sequences and the low genetic distance (4.8%) to the MRCA. Although samples in Figure 1 included very old sequences (from 1981), this low genetic distance was found only in 3 sequences (US_81NJ, ES04_LTNP_2057906 and US04_ES4) out of the 184 analyzed. The cluster viruses were unable to evolve and replicate in the different patients, but they established a productive infection. Transmission of these viruses presumably occurred because of the intravenous transmission route and probably at a time near primo-infection [46], [47].

The virological analysis carried out in this study focused, as a first approach, in *env* gene because of its role for important phenotypic characteristics. However, this not preclude that other viral genes may be associated with the lack of replication. There are other reports on mutations in accessory genes [16] or *nef* gene from the Sidney cohort [24] or in other genomic regions like the 5′non coding region [48].

**Figure 7:**
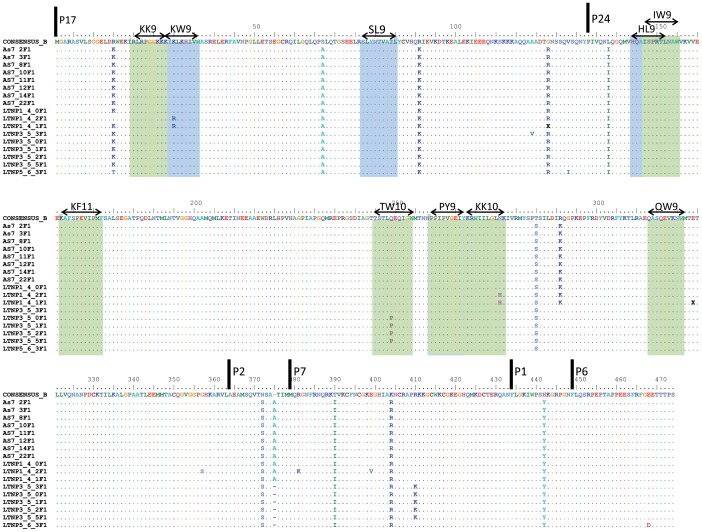
Analysis of HLA-B*57/58 and HLA-B*27 epitopes in *gag* nucleotide sequence from the cluster viruses. Amino acid sequences alignment in *gag* gene from the cluster viruses. The most important HLA-B*57/58 (blue) and HLA-B*27 (green) epitopes described in the literature are marked. Bars indicated the start position of the different proteins in HIV-1 *gag* gene. X specified mixed position in the nucleotide sequences.

The lack of evolution in the *env* sequences could reflect the immune pressure exerted by cytotoxic T-lymphocytes, in other viral genes like *gag*, *pol, nef* genes. However, analysis of the nucleotide sequences in *gag* gene revealed only the sporadic presence of mutations, but not in the HLA anchor residues of the optimally defined CTL epitopes in the viral quasispecies of patients LTNP_1 and LTNP_3 (Figure 7).

The short length (849 amino acid) and the low number of PNLGS (26–28) detected in the envelope sequences (Figure 4) also confirmed the ancestral characteristics and the lack of evolution of the cluster virus. Changes in PNLGS combined with enlargement in the hypervariable loop lengths have been related with active replication and viral evolution along the epidemic [49]–[51]. Importantly, all cluster nucleotide sequences maintained the histidine at position 12 (H12) in the SP recently identified as a signature of early infection viruses [50]. Data by other groups also detect a lack of evolution in the proviral compartment of elite patients suggesting that ongoing replication, if present, is occurring at a very low rate so as to prevent reseeding of the latent reservoir [52], [53]. All together, these results indicated that the cluster viruses showed characteristics of early epidemic viruses.

The amino acid sequences analysis identified unusual residues in the Env protein (Table 4 and Figure 5). The mutations detected in V3-C4 region, the SP and the cytoplasmic domain raised the possibility that the binding to the receptor and co-receptor and the Env expression levels may be important for the lack of evolution and low replicative capacity in the cluster virus [54]–[60]. In addition, the I322 change in the V3 region has been associated with the binding to sulfotyrosine residues within the CCR5 amino-terminal domain essential for CCR5-mediated fusion and entry [54]. K4E mutation decreases the positive charges in the SP which are associated with an increase in the rate of gp120 secretion. A higher folding rate usually leads to a decrease in the yield of correctly folded molecules [55], [56]. Mutation G226D is located in the amino acid C-terminal tail of the gp41 into the Kennedy peptide, and is implicated in cell surface Env expression, incorporation into viral particles, fusogenicity, and localization in lipid rafts [57]–[59]. This mutation also results in the V91M change, in the second coding exon of Tat protein, which is not present in any subtype B nucleotide sequence of LANL database. Although the second tat exon is largely devoid of function *in vitro*, it could be important for *in vivo* virus replication and pathogenesis [60]. The contribution of the identified residues to the lack of viral evolution is under investigation by *in vitro* mutagenesis experiments. Although, additional residues were detected in the replicating viruses of the only patient in whom we were able to rescue virus from RNA or co-culture (LTNP_1), all unusual amino acids in this region were replaced (Figure 6), either recovering the amino acid present in subtype B consensus or replaced with another unusual amino acid. These findings support the role of these residues in the low replicating phenotype. We did not observe any gross defect, stop-codons, deletions, insertions, in the *env* sequences of the cluster viruses. However, we were not able to recover the viruses by co-culture. This result is consistent with other reports, where virus co-culture was difficult in the majority of HIV-1 controllers [19], [41], [61]. Furthermore, recombinant viruses obtained with these envelopes showed a very low replicative capacity (Figure 4). Only two clones, after 14 days of culture in U87-CD4/CCR5, reached detectable, although low, levels of replication. The replication levels were 2–100 times lower than those of reference and control viruses (Figure 4). Moreover, the lack of viral replication in the cluster viruses could not be associated with the dominant presence of escape mutations in CTL epitopes in *gag* gene associated with a fitness cost [62] (Figure 7). Infection of the cluster patients with low fitness viruses has been associated with control of viral replication and disease control in LTNPs [13], [14], [21]–[23].

Several factors could be implicated in the lack of viral evolution in these patients: host genetic factors, immunological responses, and/or viral factors compromising biological fitness. In the six cluster patients, we detected an accumulation of host genetic factors associated with control of viral replication (Table 2) [2]. In addition, five of the six cluster patients had at least the HLA-B57 or B27 allele and the variant HLA-C35 (rs9264942) in homo or heterozygosis [7]. The accumulation of these protective alleles in all cluster patients is not likely to be casual. In addition, the finding that related viruses with low replication capacity were present in the cluster patients supported the role of the virus in the lack of clinical progression. We think that the extreme phenotype displayed by these patients (no clinical and virological evolution after 15–20 years of infection) is the result of a combination of host and virological factors as shown in previous studies [17], [63].

In conclusion, we identified in this report a cluster of LNTP controller patients infected by closely-related viruses with deleterious characteristics which, because of the restrictive host factors present in all patients, could not extensively replicate for the exploration of the sequence space for the replication capacity improvement. The same clinical outcome in these cluster patients, with distinct host genotypes, but infected with low replicating viruses, point to the important role of the virus in the non-progressor controller clinical phenotype.

## Supporting Information

Figure S1 **Phylogenetic analysis in the C2-V5** ***env*** **region of proviral nucleotide sequences in Spanish LTNPs and chronic patients.** The Maximum Likelihood tree was calculated with PAUP* version 4.0b incorporating the optimal evolutionary model and its parameters (Modeltest v.3.7) in a heuristic search. Box indicated nucleotide sequences of the virus cluster obtained from patients LTNP_1 (black •), LTNP_3 (red •), LTNP_5 (gray •), LTNP_RF_15 (light blue •), LTNP_RF_21 (dark blue •) and AS7 (green •). Numbers before nucleotide sequence name indicated sampling year. Black letters designated proviral nucleotide sequences obtained from LTNP patients. Red letters specified proviral nucleotide sequences obtained from chronic patients.
(TIF)

Figure S2 **Clinical and virologic follow-up from cluster patients.** Longitudinal assessment of plasma viral loads (red ▪), CD4^+^ T cell (blue ▴) and CD8+ T cell (green ▾) counts during the HIV-1 infection. Empty squares represent plasma viral load determined with a detection limit of 500 copies/ml.
(TIF)

Table S1 **Epidemiological, clinical and viral characteristics of the set 1 (Spanish patients).**
(DOC)

Table S2 **Identification of the Set 2 patients.**
(DOC)
