# Supplementary figures and images for: Identification of a Cluster of HIV-1 Controllers Infected with Low Replicating Viruses
Source: PLoS One. 2013 Oct 30;8(10):e77663. doi: 10.1371/journal.pone.0077663 (PMC3813686; doi:10.1371/journal.pone.0077663)

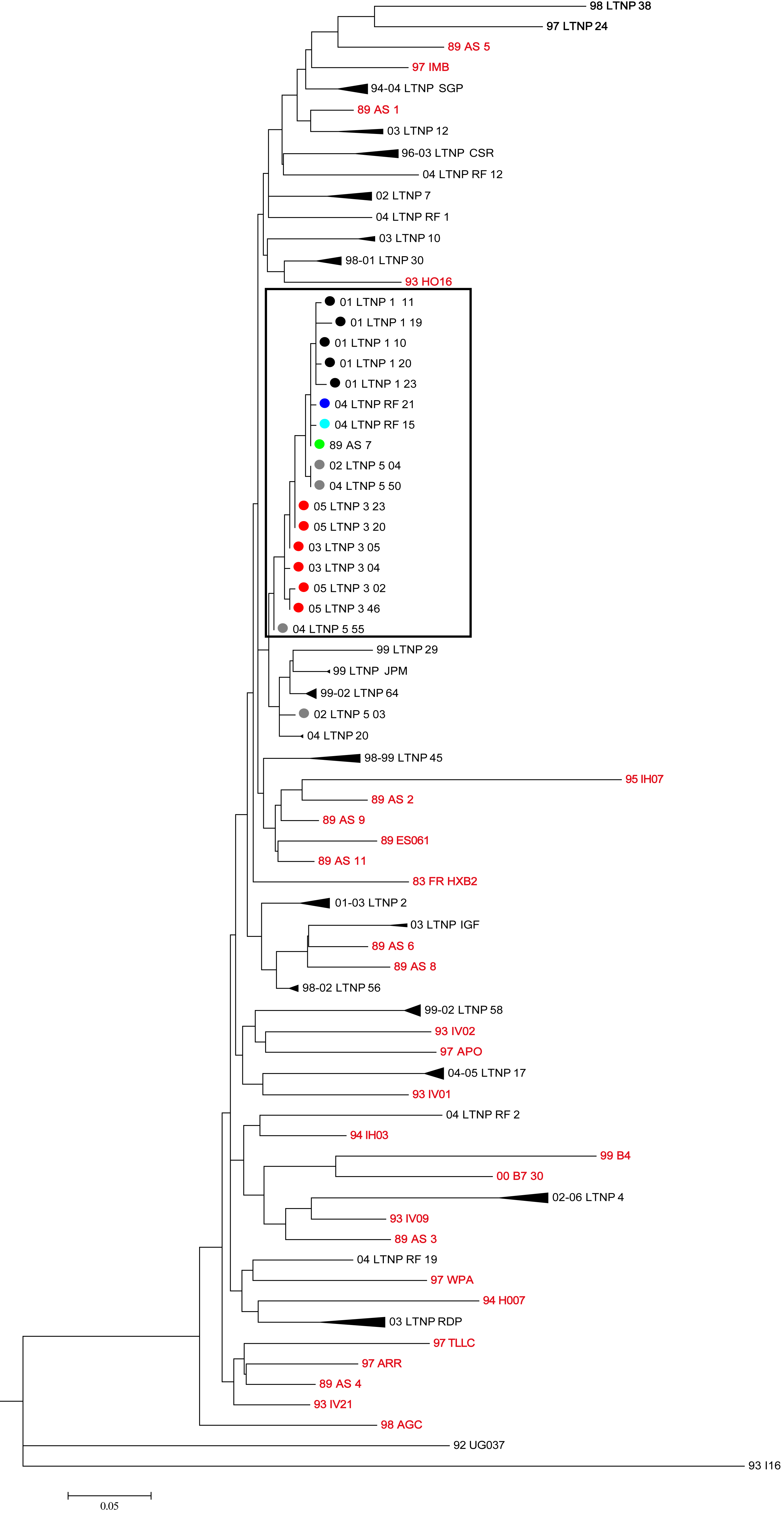

Supplement: Figure S1 — Phylogenetic analysis in the C2-V5 env region of proviral nucleotide sequences in Spanish LTNPs and chronic patients. The Maximum Likelihood tree was calculated with PAUP* version 4.0b incorporating the optimal evolutionary model and its parameters (Modeltest v.3.7) in a heuristic search. Box indicated nucleotide sequences of the virus cluster obtained from patients LTNP_1 (black •), LTNP_3 (red •), LTNP_5 (gray •), LTNP_RF_15 (light blue •), LTNP_RF_21 (dark blue •) and AS7 (green •). Numbers before nucleotide sequence name indicated sampling year. Black letters designated proviral nucleotide sequences obtained from LTNP patients. Red letters specified proviral nucleotide sequences obtained from chronic patients. (TIF) [file pone.0077663.s001.tif]

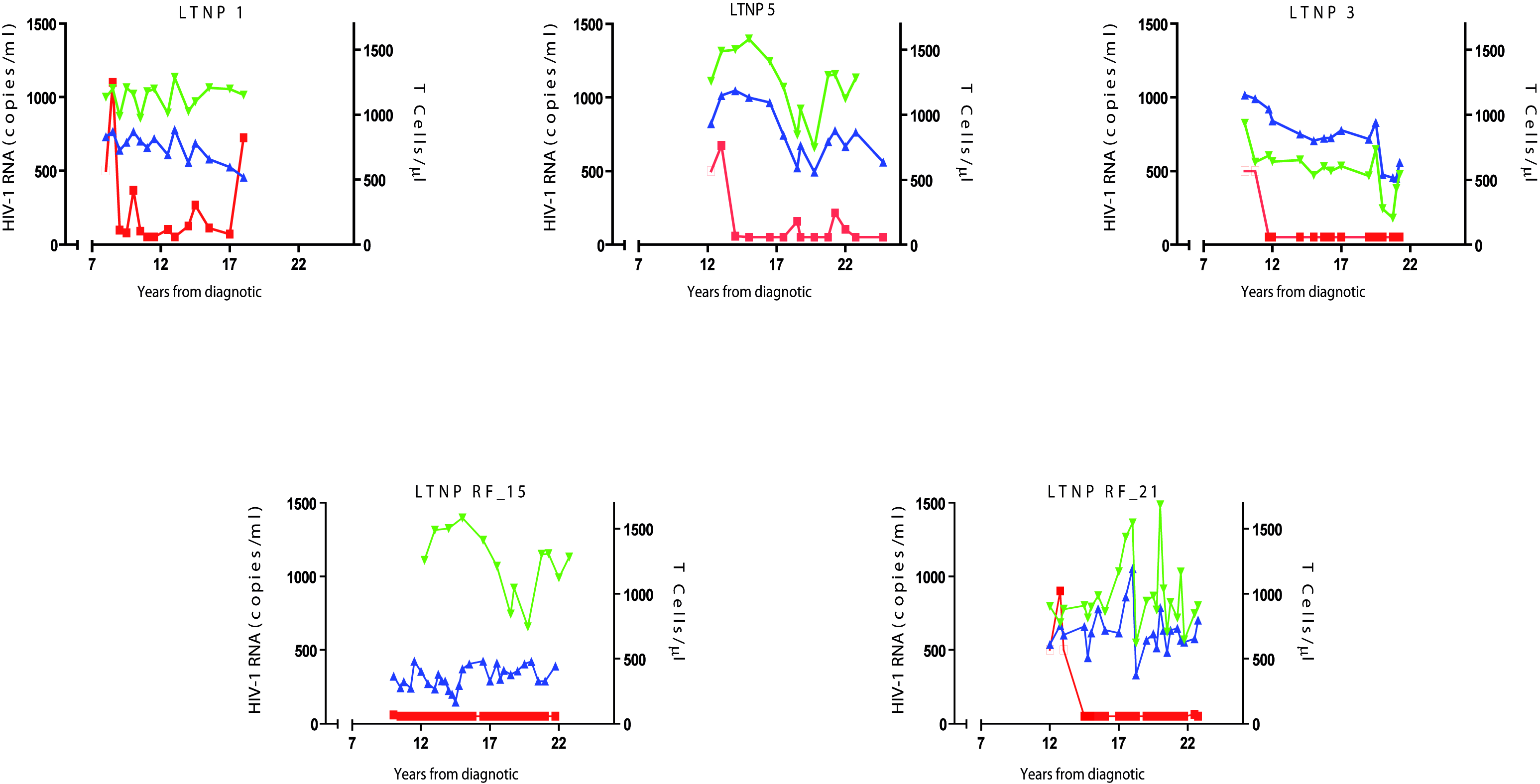

Supplement: Figure S2 — Clinical and virologic follow-up from cluster patients. Longitudinal assessment of plasma viral loads (red ▪), CD4+ T cell (blue ▴) and CD8+ T cell (green ▾) counts during the HIV-1 infection. Empty squares represent plasma viral load determined with a detection limit of 500 copies/ml. (TIF) [file pone.0077663.s002.tif]
