# Supplementary material for: Identification of a Cluster of HIV-1 Controllers Infected with Low Replicating Viruses
Source: PLoS One. 2013 Oct 30;8(10):e77663. doi: 10.1371/journal.pone.0077663 (PMC3813686; doi:10.1371/journal.pone.0077663)
Supplement: Table S1 — Epidemiological, clinical and viral characteristics of the set 1 (Spanish patients). (DOC) [file pone.0077663.s003.doc]

Tabla 1

# Table S1: Epidemiological, clinical and viral characteristics of the set 1 (Spanish patients)

| **Patient** | **Sex** | **Origen** | **Hospital** | **Transmission^a^** | **First** | **Sample** | **Clinical** | **Group** | **Mean V.Load** | **Mean CD4^+^** |
| --- | --- | --- | --- | --- | --- | --- | --- | --- | --- | --- |
|  |  |  |  |  | **HIV-1+** | **Date** | **Follow-up** |  | **(Copies/ml)** | **(Cells/µl)** |
| LTNP 2 | M | Madrid | C.S.Sandoval | IDU | 1987 | 2003 | 1995-2003 | LTNP-EC | 102 | 1125 |
| LTNP 3 | M | Madrid | C.S.Sandoval | IDU | 1988 | 2005 | 1998-2009 | LTNP-EC | <50 | 809 |
| LTNP 5 | M | Madrid | C.S.Sandoval | IDU | 1986 | 2005 | 1998-2010 | LTNP-EC | 210 | 838 |
| LTNP 20 | F | Madrid | C.S.Sandoval | IDU | 1985 | 2004 | 1990-2009 | LTNP-EC | <50 | 1099 |
| LTNP 56 | F | Madrid | C.S.Sandoval | IDU | 1989 | 2004 | 1998-2010 | LTNP-EC | <50 | 987 |
| LTNP RF 15 | M | Madrid | 12 de Octubre | IDU | 1989 | 2004 | 1999-2010 | LTNP-EC | <50 | 478 |
| LTNP RF 21 | M | Madrid | 12 de Octubre | IDU | 1985 | 2004 | 1997-2007 | LTNP-EC | <50 | 797 |
| LTNP MDM* | M | Barcelona | Germans T y P | MSM | 1988 | 2005 | 1992-2007 | LTNP-EC | <50 | 978 |
| LTNP 2057906 | M | Tarragona | Juan XXIII | HDR | 1985 | 2004 | 1997-2003 | LTNP-EC | <50 | 1191 |
| LTNP 20020753 | M | Madrid | 12 de Octubre | IDU | 1989 | 2004 | 1994-2003 | LTNP-EC | <50 | 1364 |
| LTNP 10246788 | M | Pamplona | Navarra | IDU | 1992 | 2005 | 1992-2009 | LTNP-EC | <50 | 1158 |
| LTNP 3227058 | M | Madrid | Ramón y Cajal | IDU | 1989 | 2004 | 1993-2008 | LTNP-EC | 221 | 982 |
| LTNP 357184 | M | S.Sebastian | Donostia | IDU | 1985 | 2004 | 1991-2008 | LTNP-EC | <50 | 787 |
| LTNP 3227050 | M | Madrid | Ramón y Cajal | IDU | 1989 | 2004 | 2000-2004 | LTNP-EC | <50 | 830 |
| LTNP 20044616 | M | Madrid | 12 de Octubre | IDU | 1986 | 2004 | 1998-2003 | LTNP-EC | 174 | 1060 |
| LTNP RF 3 | M | Madrid | 12 de Octubre | NK | NK | 2004 | 1996-2011 | LTNP-EC | <50 | 1331 |
| LTNP 1 | F | Madrid | C.S.Sandoval | IDU | 1990 | 2005 | 1998-2010 | LTNP-VC | 285 | 745 |
| LTNP 18122 | M | Madrid | La Princesa | IDU | 1991 | 2004 | 2000-2009 | LTNP-VC | 322 | 724 |
| LTNP 1540906 | F | Valencia | La Fe | IDU | 1995 | 2005 | 2000-2007 | LTNP-VC | 1631 | 653 |
| LTNP 279752 | F | Madrid | Carlos III | IDU | 1985 | 2005 | 1990-2005 | LTNP-VC | 254 | 548 |
| LTNP 251248 | M | Alicante | Elche | IDU | 1991 | 2004 | 1999-2005 | LTNP-VC | 836 | 584 |
| LTNP RF 9 | M | Madrid | 12 de Octubre | NK | NK | 2004 | 1997-2008 | LTNP-VC | 1313 | 576 |
| LTNP RF 12 | M | Madrid | 12 de Octubre | NK | NK | 2004 | 1997-2011 | LTNP-VC | 1965 | 747 |
| LTNP RF 19 | M | Madrid | 12 de Octubre | NK | NK | 2004 | 1998-2005 | LTNP-VC | 1326 | NK |
| LTNP 12 | M | Madrid | C.S.Sandoval | IDU | 1986 | 2004 | 2001-2010 | LTNP-NC | 2579 | 456 |
| LTNP 30 | M | Madrid | C.S.Sandoval | MSM | 1986 | 1998 | 1997-2004 | LTNP-NC | 8765 | 785 |
| LTNP 64 | M | Madrid | C.S.Sandoval | IDU | 1985 | 1999 | 1997-2006 | LTNP-NC | 12297 | 515 |
| LTNP 3227057 | M | Madrid | Ramón y Cajal | IDU | 1989 | 2004 | 2002-2007 | LTNP-NC | 2638 | 745 |
| LTNP 9684 | M | P.de Mallorca | Son Dureta | IDU | 1986 | 2005 | 1998-2003 | LTNP-NC | 2555 | 705 |
| LTNP HC2988965 | M | Tarragona | Juan XXIII | IDU | 1992 | 2004 | 1994-2003 | LTNP-NC | 5072 | 1054 |
| LTNP 357182 | M | S.Sebastian | Donostia | IDU | 1986 | 2004 | 1996-2007 | LTNP-NC | 4272 | 534 |
| LTNP 4022834 | M | P.de Mallorca | Son Dureta | MSM | 1994 | 2004 | 2000-2003 | LTNP-NC | 4115 | 910 |
| LTNP 5009564 | F | Barcelona | Clinic | HT | 1990 | 2005 | 1996-2006 | LTNP-NC | 20173 | 759 |
| LTNP 20020760 | M | Madrid | 12 de Octubre | IDU | 1992 | 2004 | 1997-2004 | LTNP-NC | 2867 | 933 |
| LTNP RF 1 | NK | Madrid | 12 de Octubre | NK | NK | 2004 | NK | NK | NK | NK |
| LTNP RF 2 | F | Madrid | 12 de Octubre | NK | NK | 2004 | 1996-2004 | LTNP-NC | 4354 | 719 |
| LTNP RF 10 | F | Madrid | 12 de Octubre | NK | NK | 2004 | 2003-2010 | LTNP-NC | 3134 | 543 |
| LTNP RF 13 | M | Madrid | 12 de Octubre | NK | NK | 2004 | 1997-2005 | LTNP-NC | 3277 | 929 |
| LTNP RF 36 | NK | Madrid | 12 de Octubre | NK | NK | 2007 | NK | NK | NK | NK |
| LTNP RF 37 | NK | Madrid | 12 de Octubre | NK | NK | 2007 | NK | NK | NK | NK |
| LTNP RF 40 | NK | Madrid | 12 de Octubre | NK | NK | 2007 | NK | NK | NK | NK |
| MH01^b^ | NK | Madrid | Carlos III | NK | 1990 | NK | NK | LTNP | NK | NK |
| MH02^b^ | NK | Madrid | Carlos III | NK | 1990 | NK | NK | LTNP | NK | NK |
| MH04^b^ | NK | Madrid | Carlos III | NK | 1987 | NK | NK | LTNP | NK | NK |
| AS1 | F | Madrid | El Patriarca | IDU | 1989 | 1989 | NK | NK | NK | NK |
| AS2 | M | Madrid | El Patriarca | IDU | 1989 | 1989 | NK | NK | NK | NK |
| AS3 | M | Madrid | El Patriarca | IDU | 1989 | 1989 | NK | NK | NK | NK |
| AS4 | M | Madrid | El Patriarca | IDU | 1989 | 1989 | NK | NK | NK | NK |
| AS5 | M | Madrid | El Patriarca | IDU | 1989 | 1989 | NK | NK | NK | NK |
| AS6 | M | Madrid | El Patriarca | IDU | 1989 | 1989 | NK | NK | NK | NK |
| AS7 | F | Madrid | El Patriarca | IDU | 1989 | 1989 | NK | NK | NK | NK |
| AS8 | M | Madrid | El Patriarca | IDU | 1989 | 1989 | NK | NK | NK | NK |
| AS9 | M | Madrid | El Patriarca | IDU | 1989 | 1989 | NK | NK | NK | NK |
| AS11 | M | Madrid | El Patriarca | IDU | 1989 | 1989 | NK | NK | NK | NK |
| S61 | M | Madrid | Gregorio Marañon | Perinatal | 1985 | 1989 | NK | NK | NK | NK |

^a^: Transmission route. IDU: intravenous drug user. MSM: Men who have Sex with Men. HT: heterosexual. HDR: hemoderivatives receptor

^b^: LTNPs describe in

* LTNP patient with double infection: two different nucleotide sequences were used from this patient. Describe in

NK: not known

1. Antoni S, Walz N, Landersz M, Humbert M, Seidl C, et al. (2007) Genetic and biological characterization of recombinant HIV type 1 with Env derived from long-term nonprogressor (LTNP) viruses. AIDS Res Hum Retroviruses 23: 1377-1386.

2. Pernas M, Casado C, Arcones C, Llano A, Sanchez-Merino V, et al. (2012) Low-replicating viruses and strong anti-viral immune response associated with prolonged disease control in a superinfected HIV-1 LTNP elite controller. PLoS One 7: e31928.
