# Supplementary material for: Identification of a Cluster of HIV-1 Controllers Infected with Low Replicating Viruses
Source: PLoS One. 2013 Oct 30;8(10):e77663. doi: 10.1371/journal.pone.0077663 (PMC3813686; doi:10.1371/journal.pone.0077663)
Supplement: Table S2 — Identification of the Set 2 patients. (DOC) [file pone.0077663.s004.doc]

Tabla 1

**Table S2:** Identification of the Set 2 of the patients

| **COUNTRY** | **SAMPLING YEAR** | **PATIENT IDENTIFICATION** | **ACCESSION NUMBER** |
| --- | --- | --- | --- |
| US | 1985 | Ba-L | AB221005 |
| US | 1986 | ADA | AF004394 |
| US | 1986 | SFMHS1 | AF025749 |
| US | 1986 | SFMHS2 | AF025750 |
| US | 1985 | SFMHS3 | AF025751 |
| US | 1986 | SFMHS4 | AF025752 |
| US | 1987 | SFMHS5 | AF025753 |
| US | 1988 | SFMHS6 | AF025754 |
| US | 1987 | SFMHS7 | AF025755 |
| US | 1986 | SFMHS8 | AF025756 |
| US | 1987 | SFMHS9 | AF025757 |
| US | 1985 | SFMHS11 | AF025758 |
| US | 1986 | SFMHS16 | AF025759 |
| US | 1986 | SFMHS17 | AF025760 |
| US | 1986 | SFMHS18 | AF025761 |
| US | 1988 | SFMHS19 | AF025762 |
| US | 1989 | SFMHS20 | AF025763 |
| US | 1985 | SFMHS21 | AF025764 |
| US | 1989 | R2 | AF128126 |
| US | 1988 | WR27 | AF286365 |
| US | 1984 | MN | AX658026 |
| US | 1990 | US1 | AY173952 |
| US | 1990 | US2 | AY173953 |
| US | 1990 | US3 | AY173954 |
| US | 1990 | US4 | AY173955 |
| US | 1990 | WEAU0575 | AY223743 |
| US | 1981 | 81CA1 | AY247218 |
| US | 1981 | 81CA2 | AY247219 |
| US | 1981 | 81GA | AY247220 |
| US | 1981 | 81NJ | AY247221 |
| US | 1981 | 81NY1 | AY247222 |
| US | 1981 | 81NY2 | AY247223 |
| US | 1981 | 81NY3 | AY247224 |
| CA | 1982 | 82CAN | AY247225 |
| US | 1984 | SF33 | AY352275 |
| US | 1985 | C | AY357338 |
| US | 1986 | JR | AY426125 |
| US | 1990 | 873 | AY713412 |
| US | 1983 | 5096 | AY835748 |
| US | 1983 | 5084 | AY835754 |
| US | 1985 | 5157 | AY835755 |
| US | 1986 | 5113 | AY835757 |
| US | 1982 | 5048 | AY835759 |
| US | 1984 | 5160 | AY835762 |
| US | 1989 | 5073 | AY835766 |
| US | 1985 | 5077 | AY835769 |
| US | 1983 | 5082 | AY835770 |
| US | 1986 | 5127 | AY835774 |
| US | 1983 | 5018 | AY835777 |
| US | 1984 | 5019 | AY835779 |
| US | 1982 | H2 | EF159970 |
| US | 1982 | H3 | EF159971 |
| US | 1982 | H5 | EF159972 |
| US | 1983 | H6 | EF159973 |
| US | 1983 | H7 | EF159974 |
| US | 1990 | BORI0637 | EF593214 |
| US | 1983 | ARV2 | K02007 |
| US | 1987 | BCSG3 | L02317 |
| US | 1985 | WMJ | M12507 |
| US | 1984 | 451 | M13137 |
| US | 1984 | SC | M17450 |
| US | 1983 | RF | M17451 |
| US | 1985 | AL-1 | M38430 |
| US | 1984 | NY5 | M38431 |
| US | 1986 | YU | M93258 |
| US | 1989 | P896 | U39362 |
| US | 1985 | WCIPR | U69584 |
| US | 1984 | SC141 | U90934 |
| NL | 1985 | ACH320 | AF069524 |
| NL | 1986 | H434 | AY970946 |
| NL | 1994 | ACH142 | DQ178989 |
| NL | 1986 | ACH19999 | EU743973 |
| NL | 1985 | ACH19542 | EU744014 |
| NL | 1987 | ACH18969 | EU744055 |
| NL | 1986 | ACH19768 | EU744097 |
| NL | 1985 | ACH19659 | EU744146 |
| GB | 1986 | GB8 | AJ271445 |
| GB | 1994 | PAT-1 | AJ535590 |
| GB | 1994 | PAT-2 | AJ535600 |
| GB | 1995 | PAT-3 | AJ535608 |
| GB | 1995 | PAT-5 | AJ535616 |
| GB | 1983 | CAM1 | D10112 |
| FR | 1983 | LAI | A04321 |
| FR | 1992 | 133 | AF041126 |
| FR | 1993 | 146 | AF041127 |
| FR | 1993 | 159 | AF041128 |
| FR | 1993 | 153 | AF041129 |
| FR | 1993 | 155 | AF041130 |
| FR | 1993 | 160 | AF041131 |
| FR | 1994 | 309 | AF041132 |
| FR | 1995 | 374 | AF041133 |
| FR | 1995 | 373 | AF041134 |
| FR | 1992 | BX08 | AY713411 |
| IT | 1995 | TRO | AY835445 |
| BE | 1990 | VI423 | DL258710 |
| BE | 1993 | VI843 | DL258715 |
| BE | 1994 | VI1399 | DQ313253 |
| BE | 1991 | SIMI | L07421 |
| DE | 1986 | D31 | U43096 |
| DE | 1986 | HAN-2 | U43141 |
| US | 2000 | ES1^a^ | EF363123 |
| US | 2004 | ES2^a^ | DQ410046 |
| US | 2004 | ES3^a^ | DQ410066 |
| US | 2004 | ES4^a^ | DQ410069 |
| US | 2004 | ES5^a^ | DQ410087 |
| US | 2004 | ES6^a^ | DQ410101 |
| US | 2005 | ES7^a^ | DQ410116 |
| US | 2004 | ES8^a^ | DQ410141 |
| US | 2004 | ES9^a^ | DQ410188 |
| US | 2004 | ES10^a^ | DQ410217 |
| US | - | BEC1^b^ | EU517815-EU517840 |
| US | - | BEC11^b^ | EU517816-EU517841 |
| US | - | BEC13^b^ | EU517817-EU517842 |
| US | - | BEC35^b^ | EU517820-EU517843 |
| US | - | BEC37^b^ | EU517821-EU517844 |
| US | - | BEC46^b^ | EU517822-EU517845 |
| US | - | BEC50^b^ | EU517824-EU517846 |
| US | - | BEC51^b^ | EU517825-EU517847 |
| US | - | BEC55^b^ | EU517827-EU517848 |
| US | - | BEC66^b^ | EU517831-EU517850 |
| US | - | BEC86^b^ | EU517834-EU517852 |
| US | - | BEC106^b^ | EU517837-EU517854 |
| US | - | BEC108^b^ | EU517835-EU517855 |
| US | - | BEC119^b^ | EU517838-EU517856 |
| US | - | BEC125^b^ | EU517839-EU517857 |

^a^:elite suppressors from

^b^:elite controllers from

REFERENCES

1. Blankson JN, Bailey JR, Thayil S, Yang HC, Lassen K, et al. (2007) Isolation and characterization of replication-competent human immunodeficiency virus type 1 from a subset of elite suppressors. J Virol 81: 2508-2518.

2. Miura T, Brockman MA, Brumme CJ, Brumme ZL, Carlson JM, et al. (2008) Genetic Characterization of Human Immunodeficiency Virus Type 1 in Elite Controllers: Lack of Gross Genetic Defects or Common Amino Acid Changes. JVirol 82: 8422-8430.
